# Subtotal laparoscopic cholecystectomy for gangrenous gallbladder during recovery from COVID-19 pneumonia

**DOI:** 10.1016/j.ijscr.2020.06.038

**Published:** 2020-06-13

**Authors:** Andrea Lovece, Emanuele Asti, Barbara Bruni, Luigi Bonavina

**Affiliations:** University of Milan, Department of Biomedical Sciences for Health, Division of General and Foregut Surgery, Italy

## Abstract

**Introduction:**

Management of acute abdomen during COVID-19 pandemic may be challenging.

**Presentation of case:**

A 42-year old man was hospitalized for Covid-19 pneumonia. Fever, respiratory symptoms and hypoxemia significantly improved over the next 2 weeks, but the patient developed abdominal pain, nausea, and low-grade fever. Computed tomography scan revealed absence of contrast enhancement of gallbladder wall and a micro-perforation of the fundus. At laparoscopy, gallbladder gangrene was confirmed and a subtotal cholecystectomy performed. Special precautions were adopted for patient transportation from the ward to a dedicated operating room, and two teams with adequate personal protective equipment took charge of the procedure. The patient was discharged home on postoperative day 7 under protective lockdown measures for 2 weeks.

**Discussion:**

The pathogenesis of acute acalcolous gangrenous cholecystitis is multifactorial. It is unknown whether a prothrombotic state induced by COVID-19 contributes to wall ischemia and perforation. Percutaneous cholecystostomy should be avoided in patients with gallbladder gangrene. Contraindications to laparoscopy are not evidence-based since aerosolization is produced during both open and laparoscopic surgical procedures. However, personal protective equipment is key for prevention.

**Conclusion:**

Early diagnosis and surgical therapy are critical in patients with gangrenous cholecystitis. Subtotal laparoscopic cholecystectomy for gangrenous gallbladder is safe and effective.

## Highlights

• Gallbladder gangrene can present during hospital stay for COVID-19.
• Protective measures are mandatory to prevent transmission to hospital personnel.
• Subtotal laparoscopic cholecystectomy is feasible, safe, and effective.

## 1 Introduction

Covid-19 has rapidly become a global pandemic with high lethality rates. Italy has been an epicenter of this outbreak, with more than 170,000 cases recorded to date from February 20th, 2020, and an estimated 13% overall mortality (www.protezionecivile.gov.it). The impact of this outbreak on the hospital health-care system has been devastating, with most resources being allocated to patients with proven or suspected infection and elective surgery canceled or delayed. There are significant implications of the pandemic also on the emergency surgery activity due to the potential spread of infection in the nosocomial environment. Surgical teams are obviously at high risk for Covid-19 exposure. The virus can survive in aerosol for at least 3 h and can be found on different surfaces for days. It is also likely that the virus can spread in smoke generated by electrocautery and ultrasonic devices. Therefore, protocols for protecting both the patient and the surgical team are mandatory. The exposure risk is potentially higher in laparoscopic surgery, given the need establish an artificial pneumoperitoneum and the consequent aerosolization of the operating room (OR) environment. It is recommended that the team is able to wear and remove safely all personal protective equipment (PPE), that traffic in and out the OR is restricted, that aerosol exposure in the OR is minimized, and that at least 30 min of air exchange between cases is allowed if negative pressure operating rooms are not available [1,2].

We present an instructive case of a Covid-19 positive patient who suffered from acute surgical abdomen during hospitalization for pneumonia and required emergency laparoscopic cholecystectomy for gangrenous, acalcolous cholecystitis.

## 2 Case presentation

The case of a 42-year-old man referred to our hospital on March 14, 2020 for acute dyspnea and fever was reviewed and reported herein according to the SCARE guidelines [3]. He had been complaining of fever and fatigue for the last 7 days, and was treated at home with amoxicillin. His past medical history and physical examination were unremarkable. Body mass index was 28. Body temperature was 375 °C, respiratory rate 20/min, and heart rate 105 beats/min. Peripheral blood saturation was 92% on air. Blood pressure was 130/95 mmHg, and the ECG showed normal sinus rhythm. Blood gas analysis under oxygen therapy 4 L/min showed pH 7,41, pCO2 426 mmHg, pO2 955 mmHg, sO2 97%. Laboratory blood tests showed WBC 5,6 × 10^3^/μL, Hb 127 g/dL, platelets 226 × 10^3^/μL, CRP 139 mg/dL, procalcitonin 0.11 ng/mL, d-dimer 0.6 u/L. A chest film showed bilateral pulmonary opacities and thickenings, and a ground-glass opacity in the right hilum. A nasopharyngeal swab resulted positive for Covid-19. The patient was admitted to a dedicated ward, and combination therapy with hydroxychloroquine, lopinavir, ritonavir, azytromicin, and low-molecular-weight heparin was initiated. Due to progressive worsening of respiratory distress with severe hypoxemia, high-flow oxygen therapy with CPAP (PEEP 7,5 cm H2O, FiO2 60%) was necessary. Fever, respiratory symptoms and hypoxemia significantly improved over the next 2 weeks and oxygen requirement gradually decreased to 2 L/min. However, the patient suddenly developed nausea and upper quadrants abdominal pain. He was afebrile, but physical examination revealed diffuse abdominal tenderness and rebound pain. Laboratory tests showed WBC 1679 × 10^3^/μL, CRP 0,3 mg/dL, amylase 140 u/L, d-dimer 0.3 u/L.

On upper abdominal ultrasound, the gallbladder appeared distended with minimal pericholecystic fluid and a positive Murphy sign. Abdominal computed tomography (CT) showed the absence of contrast enhancement of the gallbladder and a micro-perforation of the fundus. The patient was then scheduled for emergency laparoscopic cholecystectomy.

## 3 Perioperative procedure and outcomes

**Fig. 1:**
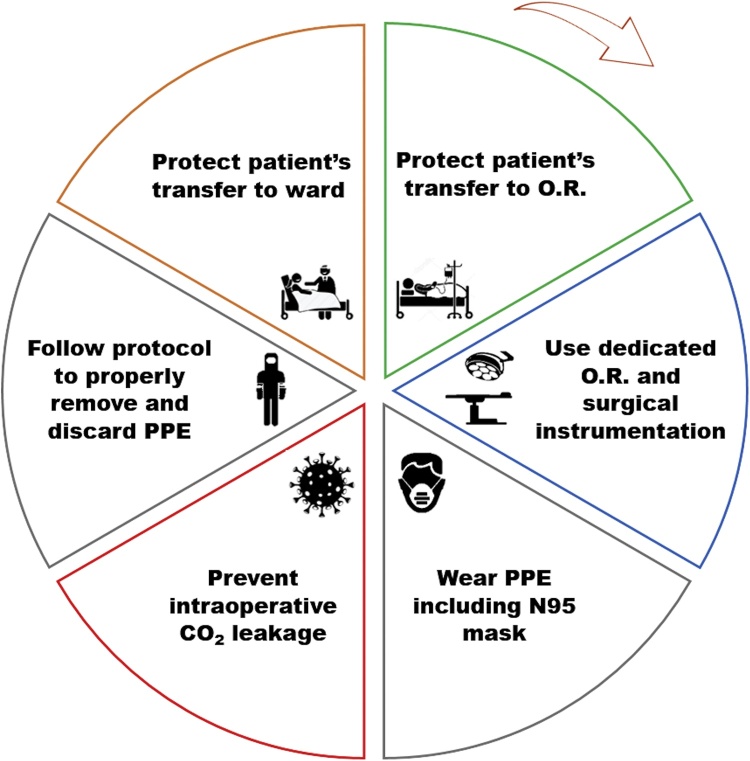
Pathway for surgical team protection.

An Internal Reviewed Board approved pathway was adopted to reduce exposure to SARS-COV-2 and to provide protection for hospital personnel (Fig. 1). Special precautions were taken for patient transportation from the ward to the OR, and back to the ward. Two teams, one inside the OR and the other outside, took charge of the procedure. A dedicated OR was used. Personal protective equipment (PPE) for the OR personnel consisted of double air cap, face shield, waterproof gown, double gloves, shoe covers, and N95 mask (Ffp2). Inside the OR, two staff surgeons, one anesthesiologist and 2 nurses were in charge of the procedure.

Rapid sequence orotracheal intubation by video-laryngoscopy was performed, and maximal care was used to minimize aerosolization and contamination throughout the surgical procedure.

**Fig. 2:**
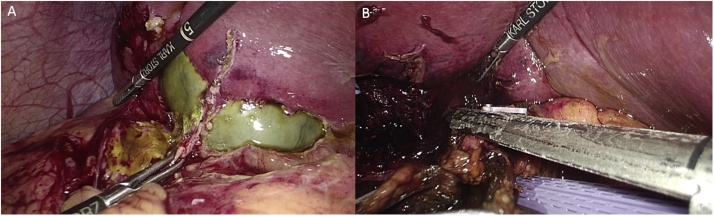
A-B. Intraoperative picture showing lysis of dense inflammatory adhesions and evidence of acute gangrenous cholecystitis (A). Intraoperative image showing stapling of the infundibulum after clipping of the cystic artery, opening of the gallbladder, and cystic duct identification. (B).

**Fig. 3:**
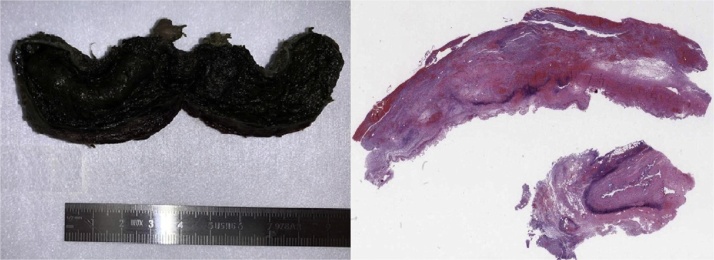
A-B. Macroscopic aspect of the gallbladder (A); microphotograph showing acute cholecystitis with extensive ulceration, full-thickness necrosis, hemorrhage, and widespread fibroblastic proliferation.

Pneumoperitoneum was established with a Veress needle. Two 12 mm and two 5 mm trocars were inserted through minimal wall incisions. Intra-operative pneumoperitoneum was set at 9 mmHg to minimize CO2 leakage without compromising exposure of the surgical field. Use of electrocautery was minimized, and smoke was aspirated through a smoke-evacuation system with filters. After lysis of dense inflammatory adhesions, a severe, gangrenous cholecystitis with a small perforation of the fundus was identified. Because a retrograde approach was deemed at risk, the gallbladder was opened and the infundibulum was transected with a stapler after identifying the cystic duct from inside (Fig. 2). The pneumoperitoneum was evacuated by the suction device before trocar removal and specimen extraction to prevent aerosol dispersal. The procedure lasted 85 min. The patient was extubated immediately after the procedure without complications. Special precautions were again adopted by the surgical team and the scrub nurse to take off the gowns and remove the face mask afterwards. Postoperative course was uneventful, and the patient was discharged home on day 5 under protective lockdown measures. Pathology confirmed transmural gallbladder necrosis (Fig. 3). At one-month follow-up the patient is doing well and two consecutive swabs resulted negative.

## 4 Comment

The clinical course of this young patient was favorable due to immediate diagnosis and surgical therapy of a gangrenous acalcolous cholecystitis. There were no specific risk factors accounting for this surgical emergency, except prolonged Covid-19 related hospitalization due to pneumonia. A recent study from China showed that 16% of confirmed COVID-19 patients present with gastrointestinal symptoms from the onset, and abdominal pain was reported by 25% of these patients [4]. By contrast, in our patient, abdominal pain manifested later during the course of the disease and the pathogenesis of cholecystitis in this context remains obscure. A possible hypothesis is that a distended gallbladder from prolonged fasting may have led to bile stasis and subsequent wall ischemia [5]. Another plausible hypothesis is that some degree of systemic inflammation [6], or immunosuppression induced by the SARS-Cov-2 infection itself or by the antiretroviral medications used to treat the disease may have contributed directly, or via an opportunistic infection, to the late onset of cholecystitis. It is unknown whether the potential subclinical coagulopathy and prothrombotic state induced by Covid-19 may have contributed to small-vessel thrombosis and ischemia of gallbladder wall [7].

Acalcolous cholecystitis results from gallbladder distension and wall ischemia. It accounts for about 10% of all cases of acute cholecystitis and typically occurs in hospitalized, critically ill, and immunosuppressed patients. Secondary infections with enteric pathogens is common, and opportunistic pathogens such as Cytomegalovirus and Criptosporidium have been identified in about half of the patients with HIV/AIDS patients [8]. Gallbladder transmural necrosis, either diffuse or focal, has been reported in 37% of patients with acute cholecystitis and 58% of patients with acalcolous cholecystitis. Risk factors associated with gangrene include older age, diabetes, fever>38 °C, tachycardia, tenderness, and increased WCC, CRP, bilirubin, urea, and creatinin [9]. Clinical manifestations of gangrenous cholecystitis may be insidious, and patients may present with mild symptoms but rapidly progress to sepsis and peritonitis [10]. A markedly distended gallbladder associated with poor wall enhancement on contrast CT scan is highly specific for gangrenous cholecystitis [11,14].

Acute abdomen secondary to gastrointestinal perforation has been reported in 4 patients with suspected COVID-19 who underwent emergency laparotomy. All patients presented pulmonary opacifications or infiltrations and three had symptoms/signs of pneumonia, but eventually tested negative for viral infection [12]. Another series reported abdominal visceral infarction in 3 COVID-19 patients, one of whom underwent open bowel resection and splenectomy [13]. A number of surgical Societies, including the American College of Surgeons (ACS), the Royal College of Surgeons of United Kingdom and Ireland (RCS), the Society of American Gastrointestinal and Endoscopic Surgeons (SAGES), the European Association for Endoscopic Surgery (EAES), and the Royal Australasian College of Surgeons (RACS) have issued guidelines in response to COVID-19 pandemic. All the above societies have warned about the potential for viral transmission during laparoscopic procedures, while the RACS has clearly stated that there is no evidence to suggest that laparoscopy puts surgical staff at higher risk of viral spread. A recent position statement issued by other Societies, including the Società Italiana di Chirurgia Endoscopica (SICE) and the World Society of Emergency Surgery (WSES), has confirmed that laparoscopic cholecystectomy “…is not more likely to spread the Covid-19 infection than open cholecystectomy” [15]. Since aerosol is generated in both open and laparoscopic surgery, common sense would suggest that proper use of personal protective equipment, dedicated patways for patient transportation, dedicated operating rooms, minimum staff, minimal use of electrocautery and high-energy devices, and routine use of smoke-evacuation systems, are key for prevention.

## 5 Conclusion

Gangrenous gallbladder is the most severe form of acalcolous cholecystitis and represents a surgical emergency. Laparoscopic cholecystectomy can safely be performed in Covid-19 patients who develop gangrenous gallbladder during hospitalization. Precautions must be taken to prevent nosocomial spread of infection and contamination of medical personnel.

## Funding

No sources of funding for the research.

## Ethical approval

The study was exempted from ethical approval by the IRB (HSD protocol file no. 0029, March 23, 2020).

## Consent

Written informed consent was obtained from the patient for publication of this case report and accompanying images. A copy of the written consent is available for review by the Editor-in-Chief of this journal on request.

## Author contribution

AL searched the literature and was a major contributor in writing the manuscript. DB was a contributor in writing and reviewing the manuscript. BB and CC performed the histopathological examination of the surgical specimen and reviewed the manuscript for important intellectual content. EA reviewed the manuscript and participated at the operation. LB operated the patient, reviewed the manuscript and was a contributor in writing it. All authors read and approved the final manuscript.

## Registration of research studies

NA.

## Guarantor

Luigi Bonavina is the guarantor.

## Provenance and peer review

Not commissioned, externally peer-reviewed.

## CRediT authorship contribution statement

**Andrea Lovece:** Data curation, Formal analysis, Writing - original draft. **Emanuele Asti:** Data curation, Writing - review & editing, Writing - original draft. **Barbara Bruni:** Data curation, Writing - review & editing, Writing - original draft. **Luigi Bonavina:** Conceptualization, Methodology, Supervision, Writing - review & editing.

## Declaration of Competing Interest

The Authors have no related conflicts of interest to declare.
